# Supplementary material for: miR-1 as a Key Epigenetic Regulator in Early Differentiation of Cardiac Sinoatrial Region
Source: Int J Mol Sci. 2024 Jun 15;25(12):6608. doi: 10.3390/ijms25126608 (PMC11204236; doi:10.3390/ijms25126608)
Supplement: Supplementary file 1 [file ijms-25-06608-s001.zip › ijms-2934910-supplementary.pdf]

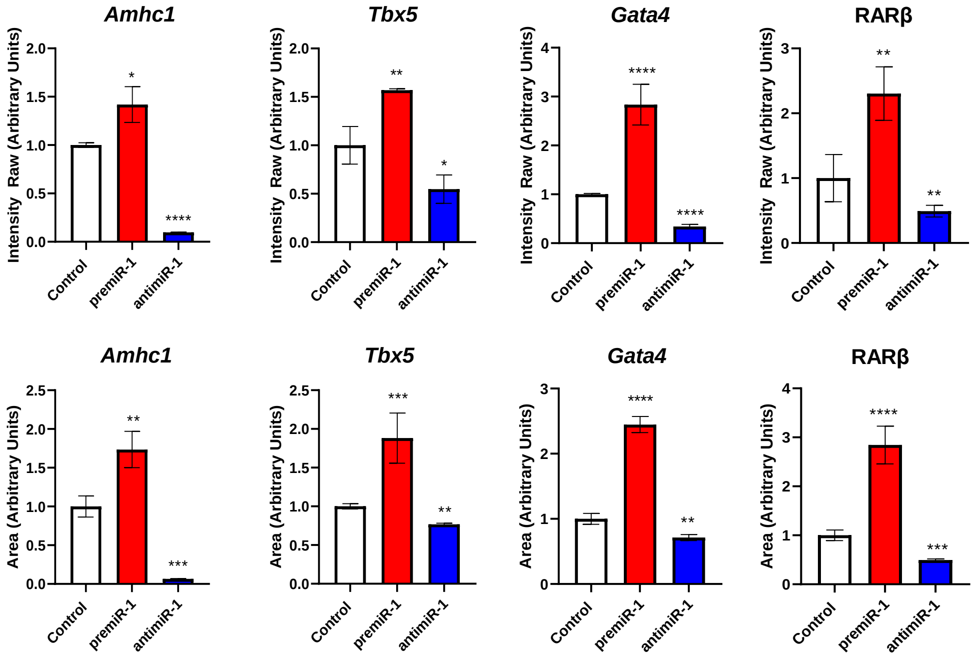

Supplementary Figure S1. Quantification of signal expression levels (upper panel) and positive area (lower panel) obtained from *Amhc1*, *Tbx5* and *Gata4* *in situ* hibridyzation and RARβ immunohistochemistry in embryos subjected to miR-1 gain- and loss-of-function experiments during early differentiation of cardiac sinoatrial region. Student's *t*-test: $*p < 0.05$, $**p < 0.01$, $***p < 0.005$, $****\ p < 0.001$ with respect to control (CFDA) embryos.

Supplementary Table S1. Effects of miR-1 gain of-function experiments during early differentiation of cardiac sinoatrial region

Microinjections into the posterior cardiac precursors	Expanded *Amhc1* expression (n)	Expanded *Tbx5* expression (n)	Expanded *Gata4* expression (n)	Expanded RARβ protein levels (n)
Control (CFDA)	0/3	0/3	0/3	0/4
premiR-1	8/8	8/8	9/9	8/8

Supplementary Table S2. Effects of miR-1 loss-of-function experiments during early differentiation of cardiac sinoatrial region

Microinjections into the posterior cardiac precursors	Decreased *Amhc1* expression (n)	Decreased *Tbx5* expression (n)	Decreased *Gata4* expression (n)	Decreased RARβ protein levels (n)
Control (CFDA)	0/3	0/3	0/3	0/4
antimiR-1	9/9	8/8	9/9	8/8

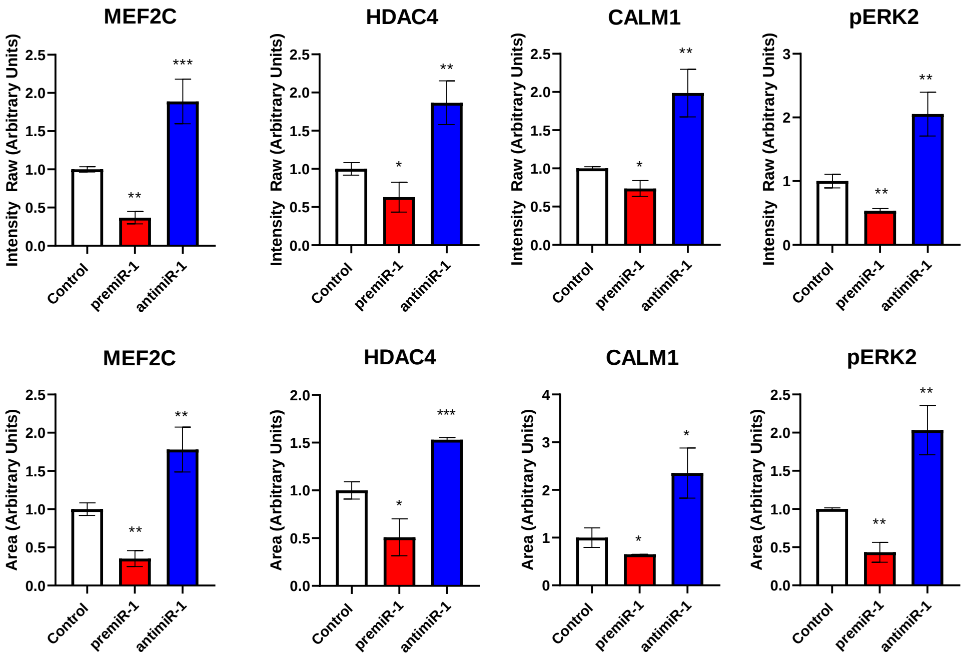

Supplementary Figure S2. Quantification of signal expression levels (upper panel) and positive area (lower panel) obtained from MEF2C, HDAC4 , CALM1/Calmodulin and pERK2 immunohistochemistry in embryos subjected to miR-1 gain- and loss-of-function experiments during early differentiation of cardiac sinoatrial region. Student's *t*-test: $*p < 0.05$, $**p < 0.01$, $***p < 0.005$ with respect to control (CFDA) embryos.

Supplementary Table S3. Effects of miR-1 gain of-function experiments during early differentiation of cardiac sinoatrial region

Microinjections into the posterior cardiac precursors	Decreased MEF2C protein levels (n)	Decreased HDAC4 protein levels (n)	Decreased CALM1 protein levels (n)	Decreased pERK2 protein levels (n)
Control (CFDA)	0/4	0/4	0/4	0/4
premiR-1	8/8	8/8	7/7	8/8

Supplementary Table S4. Effects of miR-1 loss-of-function experiments during early differentiation of cardiac sinoatrial region

Microinjections into the posterior cardiac precursors	Expanded MEF2C protein levels (n)	Expanded HDAC4 protein levels (n)	Expanded CALM1 protein levels (n)	Expanded pERK2 protein levels (n)
Control (CFDA)	0/4	0/4	0/4	0/4
antimiR-1	8/8	7/7	9/9	7/7

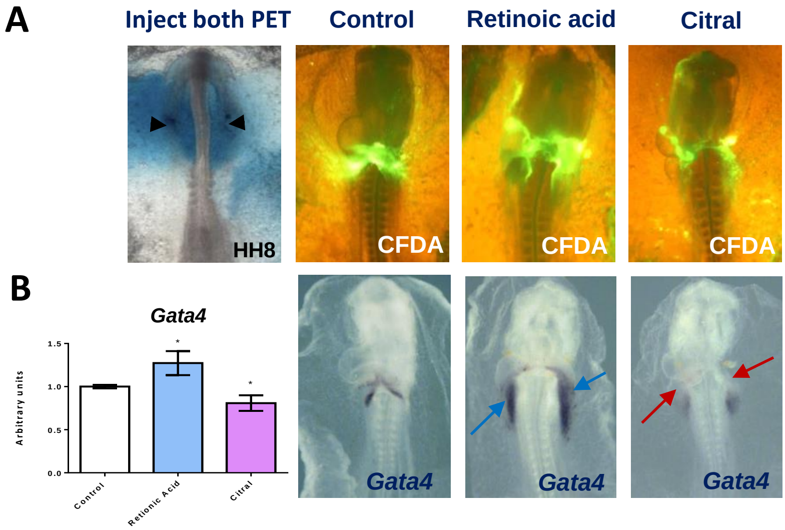

Supplementary Figure S3. A: Embryos microinjected with CFDA (control), Retinoic acid (RA) or Citral, at the level of the posterior cardiac precursors into both primitive endocardial tubes (arrowheads) , and visualization of CFDA. **B:** The gain-of-function of RA leads to expanded expression of *Gata4* in the heart tube and in the inflow tract (blue arrows). Note atrophic sinoatrial region with diminished *Gata4* expression (red arrows). RT-qPCR of RNA from dissected cardiac asa (left side) in embryos microinjected either with CFDA, RA or Citral. Standard deviations are from three independent experiments. Student's t-test: *$p < 0.05$ with respect to control (CFDA) embryos.

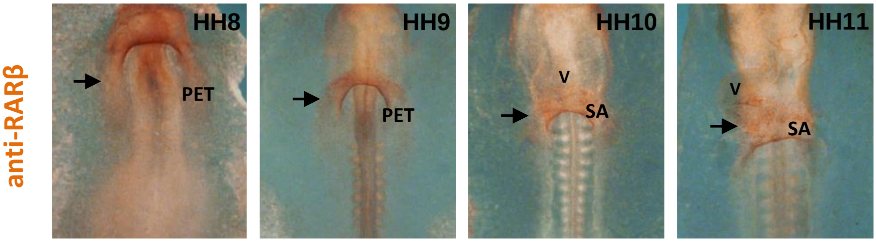

Supplementary Figure S4. Whole-mount IMH for RARβ during early chick cardiac development, from HH8 through HH11, in control embryos. Note the location of RARβ in both primitive endocardial tubes (PET), being observable in sinoatrial (SA) region at later stages. Arrows: positive staining. V: ventricle.

Chicken *Hdac4* ENST00000345617.3 3' UTR length: 370

Conserved

<table>
<tr><th></th><th>Predicted consequential pairing of target region (top) and miRNA (bottom)</th><th>Site type</th><th>Context++ score</th><th>Context++ score percentile</th><th>Weighted context++ score</th><th>Conserved branch length</th><th>P_{CT}</th></tr>
<tr><td>Position 2197-2203 of HDAC4 3' UTR
gga-miR-1a-3p</td><td>5' ...UGAAGGGGCUGUUUUCAUUCCAC...
||||||
3' AUGUAUGAAGAAAUGUAAGGU</td><td>7mer-A1</td><td>-0.04</td><td>30</td><td>-0.03</td><td>4.013</td><td>0.62</td></tr>
<tr><td>Position 2287-2293 of HDAC4 3' UTR
gga-miR-1a-3p</td><td>5' ...UACUUUCGAUACGGAACAUUCCU...
|||||||
3' AUGUAUGAAGAAAUGUAAGGU</td><td>7mer-m8</td><td>-0.18</td><td>73</td><td>-0.13</td><td>4.675</td><td>0.77</td></tr>
<tr><td>Position 2320-2326 of HDAC4 3' UTR
gga-miR-1a-3p</td><td>5' ...UCUCAGUCAUGUAUUCAUUCCAU...
||||||
3' AUGUAUGAAGAAAUGUAAGGU</td><td>7mer-A1</td><td>-0.09</td><td>54</td><td>-0.06</td><td>4.784</td><td>0.66</td></tr>
<tr><td>Position 2197-2203 of HDAC4 3' UTR
gga-miR-1b-3p</td><td>5' ...UGAAGGGGCUGUUUUCAUUCCAC...
||||||
3' AUGUAUGAAGAAUUGUAAGGU</td><td>7mer-A1</td><td>-0.04</td><td>30</td><td>-0.03</td><td>4.013</td><td>0.62</td></tr>
<tr><td>Position 2287-2293 of HDAC4 3' UTR
gga-miR-1b-3p</td><td>5' ...UACUUUCGAUACGGAACAUUCCU...
|||||||
3' AUGUAUGAAGAAUUGUAAGGU</td><td>7mer-m8</td><td>-0.18</td><td>73</td><td>-0.13</td><td>4.675</td><td>0.77</td></tr>
<tr><td>Position 2320-2326 of HDAC4 3' UTR
gga-miR-1b-3p</td><td>5' ...UCUCAGUCAUGUAUUCAUUCCAU...
||||||
3' AUGUAUGAAGAAUUGUAAGGU</td><td>7mer-A1</td><td>-0.09</td><td>54</td><td>-0.06</td><td>4.784</td><td>0.66</td></tr>
</table>

Human *Hdac4* ENST00000345617.3 3' UTR length: 4929

Conserved

<table>
<tr><th></th><th>Predicted consequential pairing of target region (top) and miRNA (bottom)</th><th>Site type</th><th>Context++ score</th><th>Context++ score percentile</th><th>Weighted context++ score</th><th>Conserved branch length</th><th>P_{CT}</th></tr>
<tr><td>Position 2334-2340 of HDAC4 3' UTR
hsa-miR-1-3p</td><td>5' ...UGAAGCCACCAGUUUCAUUCCAA...
||||||
3' UAUGUAUGAAGAAAUGUAAGGU</td><td>7mer-A1</td><td>-0.04</td><td>57</td><td>-0.03</td><td>4.013</td><td>0.62</td></tr>
<tr><td>Position 3514-3520 of HDAC4 3' UTR
hsa-miR-1-3p</td><td>5' ...UUCUUUUUGAUCAGAACAUUCCU...
|||||||
3' UAUGUAUGAAGAAAUGUAAGGU</td><td>7mer-m8</td><td>-0.14</td><td>83</td><td>-0.10</td><td>4.675</td><td>0.77</td></tr>
<tr><td>Position 3547-3553 of HDAC4 3' UTR
hsa-miR-1-3p</td><td>5' ...UCACAGCCACGUGCUCAUUCCAU...
||||||
3' UAUGUAUGAAGAAAUGUAAGGU</td><td>7mer-A1</td><td>-0.08</td><td>71</td><td>-0.06</td><td>4.784</td><td>0.66</td></tr>
</table>

Supplementary Figure S5. Bioinformatic prediction between miR-1 and *Hdac4* in Chicken and Human. The overlapping target gene was predicted through TargetScan. Context++ score and features that contribute to the context++ score are evaluated as in Agarwal et al. [109]. Conserved branch lengths and PCT are evaluated as in Friedman et al. [110], with an expanded 46-species alignment as described in Agarwal et al. [109].

Chicken *Calm1* ENST00000356978.4 3' UTR length: 117

Conserved

	Predicted consequential pairing of target region (top) and miRNA (bottom)	Site type	Context++ score	Context++ score percentile	Weighted context++ score	Conserved branch length	P_{CT}
Position 828-834 of CALM1 3' UTR<br>gga-miR-1a-3p	5' ...AUUACAAUUUAUGUCCAUUCCAA...<br>|||||| 3' AUGUAUGAAGAAAUGUAAGGU	7mer-A1	-0.17	71	-0.02	7.004	0.68
Position 828-834 of CALM1 3' UTR<br>gga-miR-1b-3p	5' ...AUUACAAUUUAUGUCCAUUCCAA...<br>|||||| 3' AUGUAUGAAGAAUUGUAAGGU	7mer-A1	-0.17	71	-0.02	7.004	0.68

Human Calm1 ENST00000356978.4 3' UTR length: 3558

Conserved

	Predicted consequential pairing of target region (top) and miRNA (bottom)	Site type	Context++ score	Context++ score percentile	Weighted context++ score	Conserved branch length	P_{CT}
Position 830-836 of CALM1 3' UTR<br>hsa-miR-1-3p	5' ...AUUACAGUUUACGUCCAUUCCAA...<br>|||||| 3' UAUGUAUGAAGAAAUGUAAGGU	7mer-A1	-0.17	86	-0.17	7.004	0.68

Supplementary Figure S6. Bioinformatic prediction between miR-1 and *Calm1/Calmodulin* in Chicken and Human. The overlapping target gene was predicted through TargetScan. Context++ score and features that contribute to the context++ score are evaluated as in Agarwal et al. [109]. Conserved branch lengths and PCT are evaluated as in Friedman et al. [110], with an expanded 46-species alignment as described in Agarwal et al. [109].

Chicken *Mapk1* ENST00000215832.6 3' UTR length: 3656

Conserved

<table>
<tr><th></th><th>Predicted consequential pairing of target region (top) and miRNA (bottom)</th><th>Site type</th><th>Context++ score</th><th>Context++ score percentile</th><th>Weighted context++ score</th><th>Conserved branch length</th><th>P_{CT}</th></tr>
<tr><td>Position 2420-2427 of MAPK1 3' UTR
gga-miR-1a-3p</td><td>5' ...CAGUUGACUGUCUAAACAUUCCA...
||| |||||||
3' AUGUAUGAAGAAA-UGUAAGGU</td><td>8mer</td><td>-0.25</td><td>81</td><td>0.00</td><td>7.839</td><td>0.96</td></tr>
<tr><td>Position 2420-2427 of MAPK1 3' UTR
gga-miR-1b-3p</td><td>5' ...CAGUUGACUGUCUAAACAUUCCA...
||| |||||||
3' AUGUAUGAAGAAU-UGUAAGGU</td><td>8mer</td><td>-0.25</td><td>81</td><td>0.00</td><td>7.839</td><td>0.96</td></tr>
</table>

Human *Mapk1* ENST00000215832.6 3' UTR length: 9750

Conserved

<table>
<tr><th></th><th>Predicted consequential pairing of target region (top) and miRNA (bottom)</th><th>Site type</th><th>Context++ score</th><th>Context++ score percentile</th><th>Weighted context++ score</th><th>Conserved branch length</th><th>P_{CT}</th></tr>
<tr><td>Position 2746-2753 of MAPK1 3' UTR
hsa-miR-1-3p</td><td>5' ...AAAAUUCCUGCUGAAACAUUCCA...
|||||||
3' UAUGUAUGAAGAAAUGUAAGGU</td><td>8mer</td><td>-0.26</td><td>94</td><td>-0.11</td><td>7.839</td><td>0.96</td></tr>
</table>

Supplementary Figure S7. Bioinformatic prediction between miR-1 and *Erk2/Mapk1* in Chicken and Human. The overlapping target gene was predicted through TargetScan. Context++ score and features that contribute to the context++ score are evaluated as in Agarwal et al. [109]. Conserved branch lengths and PCT are evaluated as in Friedman et al. [110], with an expanded 46-species alignment as described in Agarwal et al. [109].

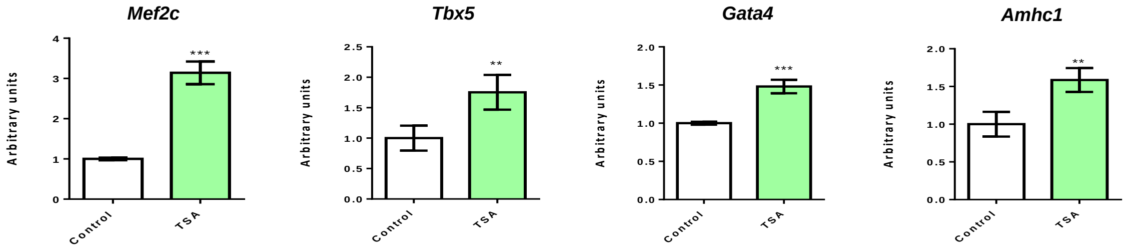

Supplementary Figure S8. RT-qPCR of RNA from dissected cardiac *asa* (right side) in embryos microinjected either with CFDA, or TSA (histone deacetylase –HDAC- inhibitors) at the level of the posterior cardiac precursors into both primitive endocardial tubes. Treatment with TSA leads to increased *Mef2c*, *Tbx5*, *Gata4* and *Amhc1* transcripts. Student's t-test: $^{**}p < 0.01$, $^{***}p < 0.005$ with respect to control (CFDA) embryos.

qPCR primers		
Gene	Specie	Sequence
Amhc1_Forward	Gallus Gallus	AAGACCGGAAGAACATGGTG
Amhc1_Reverse	Gallus Gallus	GAGACTCAGCCATGTCAGCA
Tbx5_Forward	Gallus Gallus	AACCCCTACCCGATCTCCCC
Tbx5_Reverse	Gallus Gallus	CCATGTAGGGCTTCTTGTAGGG
Gata4_Forward	Gallus Gallus	TCAACTGTGGGGCCATGTC
Gata4_Reverse	Gallus Gallus	GCATTACAGACGGGCTCTCC
Mef2c_Forward	Gallus Gallus	CCCCAGGAATGCTGTCTTTA
Mef2c_Reverse	Gallus Gallus	AGTGGAATTCATTCCGGTGA
Raldh2_Forward	Gallus Gallus	ACTGAGATTTAAAACGGTGGATGA
Raldh2_Reverse	Gallus Gallus	TCTCTCTACCATTGCCAGACA
CrabpI_Forward	Gallus Gallus	CCACCTGGGAGAATGAAAAC
CrabpI_Reverse	Gallus Gallus	CATTCTACTGGGGCTTTCCA
CrabpII_Forward	Gallus Gallus	TCAAGACCTCAACCACCGTG
CrabpII_Reverse	Gallus Gallus	GATGAGCTCCCCATCGTTGG
Rarα_Forward	Gallus Gallus	AGGTGGACAAACTGCAGGAG
Rarα_Reverse	Gallus Gallus	CGGGATCTCCATCTTCAGTG
Rarβ_Forward	Gallus Gallus	CTGTAAAAGGTCGATGAAGTCT
Rarβ_Reverse	Gallus Gallus	TTTTATCTTTTACGTTTGAAAACTTT
Rarϒ_Forward	Gallus Gallus	ATGTTCCCCAGAATGCTGAT
Rarϒ_Reverse	Gallus Gallus	GGCTCCTCCTCGAACATCTC
Rxrα_Forward	Gallus Gallus	GGCTGAAGTGGAGGCATTAC
Rxrα_Reverse	Gallus Gallus	AAGAGGTGCTCCAGGCATTT
Rxrϒ_Forward	Gallus Gallus	GGAAGCCTACACGAAGCAGA
Rxrϒ_Reverse	Gallus Gallus	GGTGTCTCCAGCATCTCCAT
Hdac4_Forward	Gallus Gallus	ATGGCATCTCTGTCGGTAGG
Hdac4_Reverse	Gallus Gallus	CATGACAGGCAGGAGTA
Calm1/Calmodulin_Forward	Gallus Gallus	TATCAGTGCAGCAGAGCTACG
Calm1/Calmodulin_Reverse	Gallus Gallus	GGAGGTCTCTTCACTTTGCAG
Erk2/Mapk1_Forward	Gallus Gallus	GAGCATCAGACGTACTGCCA
Erk2/Mapk1_Reverse	Gallus Gallus	AATGTGGTCGTTGCTGAGGT
Cripto_Forward	Gallus Gallus	TGGCTGTGTCGGTGTCTCTA
Cripto_Reverse	Gallus Gallus	AGGTGAGAAGGCAAATCAGC
Gapdh_Forward	Gallus Gallus	TGTCCTCTCTGGCAAAGTCC
Gapdh_Reverse	Gallus Gallus	TGCCCATTGATCACAAGTTT

Supplementary Table S5. Primer sequences and corresponding genes for qRT-PCR (Gallus gallus).

3'UTRs cloning primers		
**Hdac4_3'UTR_F position 1**	Gallus Gallus	GGAAGCTTCAGCCACCTCACCACTTACA
**Hdac4_3'UTR_R position 1**	Gallus Gallus	GGACTAGTTAGAGGGAGAACCAGCTCCA
**Hdac4_3'UTR_F position 2**	Gallus Gallus	GGAAGCTTCCCTCCTGCAGGTTCTCTAA
**Hdac4_3'UTR_R position 2**	Gallus Gallus	GGACTAGTGGGTCACATGGGGTTGATAC
**Calm1_3'UTR_F**	Gallus Gallus	GGACTAGTCACTTGGTGCATGGCTTAGA
**Calm1_3'UTR_R**	Gallus Gallus	GGAAGCTTTGCATCAGTTGAGAGCAACA
**Erk2/Mapk1_3'UTR_F**	Gallus Gallus	GGACTAGTAAACCATTTGGCCAGAAGAA
**Erk2/Mapk1_3'UTR_R**	Gallus Gallus	GGAAGCTTGAGCATTTGAAGCCATGTGA
**Mut_Hdac4_3'UTR_F position 1**	Gallus Gallus	CGATACGGAAGATACCTTT
**Mut_Hdac4_3'UTR_R position 1**	Gallus Gallus	AAAGGTATCTTCCGTATCG
**Mut_Hdac4_3'UTR_F position 2**	Gallus Gallus	CATTCCATAGAAGACTTGCTTG
**Mut_Hdac4_3'UTR_R position 2**	Gallus Gallus	CAAGCAAGTCTTCTATGGAATG
**Mut_Calm1_3'UTR_F**	Gallus Gallus	TTTATGTCCATAGCAAGTTGT
**Mut_Calm1_3'UTR_R**	Gallus Gallus	ACAACTTGCTATGGACATAAA
**Mut_Erk2/Mapk1_3'UTR_F**	Gallus Gallus	CTTGCAGCACATCCCCCTTT
**Mut_Erk2/Mapk1_3'UTR_R**	Gallus Gallus	AAAGGGGGATGTGCTGCAAG

Supplementary Table S6. Primer sequences 3'UTR and site-directed mutagenesis 3'UTR (Mut) corresponding *Hdac4* (position 1 and position 2); *Calm1/Calmodulin* and *Erk2/Mapk1* genes for luciferase assays (Gallus gallus).
